# Difference in Procedure-Related Risk of Miscarriage between Early and Mid-Trimester Amniocentesis: A Retrospective Cohort Study

**DOI:** 10.3390/diagnostics11061098

**Published:** 2021-06-16

**Authors:** Kelly Steinfort, Ellen Van Houtven, Yves Jacquemyn, Bettina Blaumeiser, Philip Loquet

**Affiliations:** 1 Department of Obstetrics and Gynecology, Antwerp University Hospital, UZA, ASTARC Antwerp University, 2610 Wilrijk, Belgium; Ellen.vanhoutven@student.uantwerpen.be (E.V.H.); Yves.Jacquemyn@uza.be (Y.J.); 2 Department of Global Health, Antwerp University Hospital, UZA, 2610 Wilrijk, Belgium; 3 Department of Medical Genetics, Antwerp University Hospital, UZA, University of Antwerp, 2610 Wilrijk, Belgium; Bettina.blaumeiser@uantwerpen.be; 4 Department of Obstetrics and Gynecology, St Augustinus Hospital, 2610 Wilrijk, Belgium; Philip.loquet@gmail.com

**Keywords:** early amniocentesis, mid-trimester amniocentesis, prenatal diagnosis, miscarriage, procedure-related fetal loss, pregnancy loss

## Abstract

Early amniocentesis (EA)—before 15 gestational weeks—is not recommended because of a high rate of miscarriages. Most studies performed amniocentesis at very early stages of pregnancy (11–13 weeks of gestational age). However, amniocentesis performed at 14 gestational weeks could be an important alternative to mid-trimester amniocentesis (MA) because it shortens the time period between the screening (non-invasive prenatal test (NIPT)) and the diagnostic test (amniocentesis). This study aimed to compare the procedure-related risk of miscarriage between MA (15 + 0 to 17 + 6 weeks of gestational age) and EA (14 + 0–6 weeks of gestational age). This is a multicentric, retrospective cohort study from 1 January 2007 to 21 November 2018, comparing the MA to the EA cohort. Procedure-related fetal loss is defined as spontaneous abortion occurring within 4 weeks of the procedure. Multiple gestations, amniocenteses performed after 17 or before 14 weeks, indications other than prenatal genetic diagnoses and procedures performed by less experienced gynaecologists were excluded. Complete outcome data were available for 1107 out of 1515 women (73.1%): 809 (69.9%) in the MA and 298 (83.2%) in the EA cohort. No significant difference was found (EA 0.82% vs. MA 0.36%; *p* = 0.646). The difference was 0.46% (odds ratio = 0.673; 95% confidence interval = 0.123–3.699). This study found no significant difference in the procedure-related risk of miscarriage when EA was compared to MA. EA might be considered a safe alternative, though further research is necessary.

## 1. Introduction

Amniocentesis is the most commonly performed invasive prenatal diagnostic technique offered in the second trimester of pregnancy to pregnant women with an increased risk of chromosomal abnormalities. It is usually preformed at 15 gestational weeks and is called mid-trimester amniocentesis (MA). The genetic results are available within 7 days. The most serious complication of this invasive procedure is possible miscarriage or fetal loss [1]. Several studies have proposed miscarriage rates, but no consensus has been found. A randomised controlled trial (RCT) was conducted by Tabor et al. in 1989, and its miscarriage rate of 1% is considered the gold standard [2,3]. However, more recent studies have shown significantly lower percentages of procedure-related fetal losses [4].

Chorionic villus sampling (CVS) is a first-trimester alternative for prenatal diagnosis. Mostly it is performed between 11–13 weeks of gestation. Placental villi may be obtained through transcervical or transabdominal access to the placenta [1,2,5,6,7,8,9]. The primary advantage of CVS over AC is that the procedure can be performed earlier in pregnancy after an abnormal first-trimester ultrasound examination. This allows for more and quicker management decisions [1,2,5,6,7,8,9]. Although according to the literature AC shows more accurate results in comparison to CVS, this is because of the phenomenon called confined placental mosaicism, which gives rise to false-positive results [1,3,4,5,6,10]. It is seen in 1% of the samples retrieved with CVS and in 0.25% with AC. When mosaicism is found by CVS, AC is offered to asses if the mosaicism is present in amniocytes [1,2,5,6,7,8,9].

When amniocentesis is performed before 15 gestational weeks, it is called early amniocentesis (EA) [3]. This shortens the time period between the screening test at 11–14 weeks (non-invasive prenatal test, NIPT) and the diagnostic test (amniocentesis) that needs to be conducted to confirm a positive screening test [11]. The greatest advantage is that it reduces parents’ psychological distress in this uncertain period and allows for more optimal pregnancy management and feto-maternal bonding [12]. It could be offered as an alternative to CVS in the first trimester.

The literature advises against EA because it is associated with an increased risk of fetal loss and talipes equinovarus [1,2,5,6,7,8,9]. Unfortunately, the procedures were performed at very early stages of pregnancy (11–13 weeks of gestational age). However, to our knowledge, EA at 14 gestational weeks has never been investigated. The primary purpose of this study was to compare the procedure-related risk of miscarriage posed by EA performed at 14 gestational weeks to MA at 15–17 gestational weeks.

## 2. Materials and Methods

A multicentric, retrospective cohort study was performed on pregnant women with singleton pregnancy who underwent amniocentesis at the Antwerp University Hospital or St Augustinus Hospital from 1 January 2007 to 21 November 2018. The exclusion criteria were multiple pregnancies, amniocentesis performed after 17 or before 14 gestational weeks, indications other than prenatal genetic diagnoses and procedures performed by less-experienced gynaecologists (defined as those other than Drs P.L., Y.J. and L.Y.).

The protocol was reviewed and approved by each of the two participating centres’ ethical boards. (approval codes: 170707MASTER, 17/20/244, Date: 11–24 July 2017)

**Figure 1:**
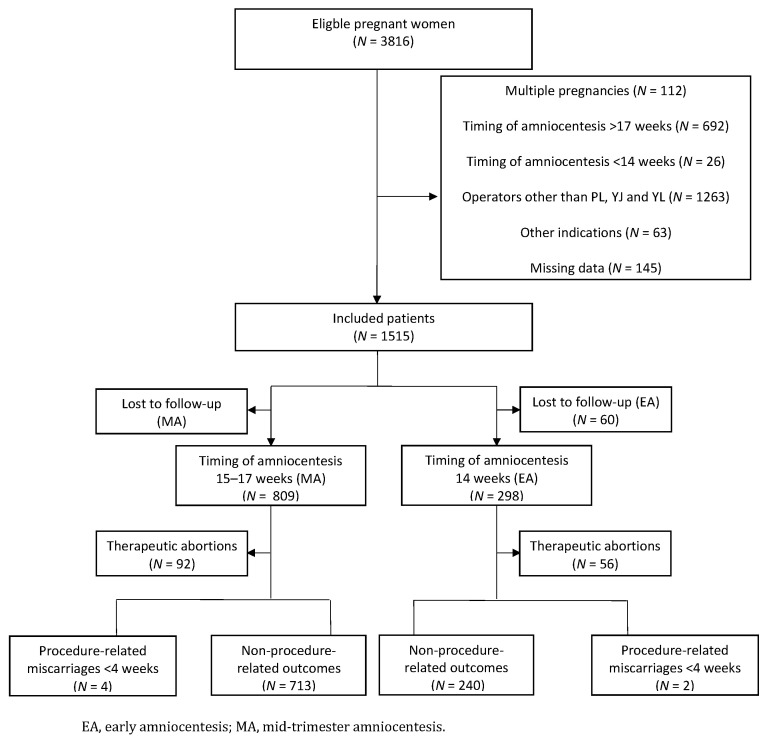
Flowchart of the study population.

The required sample size was based on the gold-standard RCT from 1998 [8], which also compared the procedure-related risk of fetal loss from EA to MA and on a more recent RCT by Wilson et al. [13]. On the basis of a non-inferiority study design, the miscarriage rate of EA could not be higher than that of MA. One-sided alpha-value was kept at 0.05 and power at 0.80. A sample size of 540 patients was used for this study, with 270 patients in each cohort. The trial profile is displayed in Figure 1.

All procedures were performed following a standardised method: antiseptic transabdominal freehand needle insertion was done under continuous ultrasonic colour Doppler guidance using a 22-gauge spinal needle. The three operators had the same amount of experience, and each one of them had performed more than 100 amniocenteses. Studies have shown that the risk of miscarriage, failed attempts and maternal contamination decreases with more extensive experience of the operator [1,3,4,5,6,10].

Given the retrospective study design, information was also collected following a standard procedure using an electronic database. The following data were collected for all the patients:

- Timing of the procedure (EA at 14 and MA at 15–17 gestational weeks)
- Final pregnancy outcome (procedure-related miscarriages <4 weeks after amniocentesis, non-procedure-related outcomes, live births and therapeutic abortions)
- Indication of amniocentesis (ultrasound abnormality, positive serum screening, abnormal first-trimester screening, genetic disorder in family history, genetic disorder in previous pregnancy, maternal age, personal request, abnormal NIPT and abnormal chorionic villus sampling)
- Genetic results (trisomy 21, 18, 13 or others)
- Maternal age

When the timing of the procedure was unknown, the patient was classified as ‘missing data’ and excluded. If the primary outcome was not obtained, the patient was classified as ‘lost to follow-up’.

Statistical calculations were performed using SPSS (v.25; IBM Corp, Armonk, NY, USA). This study aimed to investigate the difference between the miscarriage rates of MA (15–17 weeks) and EA (14 weeks). For this purpose, a Fisher’s exact test was performed, and odds ratios with a 95% confidence interval (CI) were calculated. Secondary analysis was conducted to compare the two groups on baseline characteristics: median age, indication for amniocentesis and genetic results using a Student’s *t*-test for continuous variables and a chi-square test for categorical variables. The missing data were excluded by SPSS.

## 3. Results

A total of 3824 pregnant women were identified who underwent amniocenteses between 1 January 2007 and 21 November 2018. Of the total, 2309 women were excluded on the basis of the exclusion criteria mentioned in the “Methods” section. (See the trial profile shown in Figure 1.) There were 1515 eligible women. Of this, complete information was known for 1107 (73.1%): 809 (69.9%) in the MA cohort and 298 (83.2%) in the EA cohort. Four hundred eight (26.9%) women were lost to follow-up because the primary outcome was unknown.

The gestational age at the time of amniocentesis is summarised in Table 1. For the EA group, the mean gestation was 14 + 4 weeks. For the MA group, it was 15 + 6 weeks.

Table 2 details the patient characteristics for both groups. The mean maternal age at entry was similar in both groups: 33.559 and 33.557 years in the MA and EA groups, respectively. A significant difference was seen in the indication for undergoing amniocentesis. In the MA cohort, it was mostly performed for abnormal ultrasound findings (16.56%), positive serum screening (30.53%) and maternal age (26.21%). The same results were seen in the EA cohort. However, noticeably more amniocentesis was performed for abnormal NIPT results: 11.41% in the EA group in comparison to 4.2% in the MA group. Remarkably, only 73.52% (MA) and 61.76% (EA) of the abnormal NIPT results could be confirmed by amniocentesis.

Chromosomal abnormalities were identified in 60 cases of EA and 89 cases of MA. A significant difference was found in the genetic results between the two groups. For the MA group, the following diagnoses were identified: 46 trisomy 21 (5.69%), 1 trisomy 13 (0.14%), 5 trisomy 18 (0.62%) and 37 other genetic abnormalities (4.57%). In the EA group, there were 38 trisomy 21 cases (12.75%), 1 trisomy 13 (0.34%), 2 trisomy 18 (0.67%) and 9 other genetic abnormalities (6.36%). The last group included balanced translocations, sex chromosome aneuploidies (Turner’s and Klinefelter’s syndromes), and different deletions and duplications. Not all women with chromosomal anomalies underwent therapeutic abortions. In the EA group, 45 of the 60 (75.00%) women chose to have an abortion. In the MA group, 61 out of the 89 (68.45%) women chose to terminate the pregnancy. In total, 71.1% of the women underwent an abortion for chromosomal abnormalities: 91.7% of trisomy 21, 100.0% of trisomy 13, 85.7% of trisomy 18 and 37.5% of other genetic anomalies. Therapeutic abortions were deducted from non-procedure-related outcomes because abortions were performed in the time-frame before a procedure-related miscarriage could occur.

Total procedure-related pregnancy losses (miscarriage < 4 weeks after the procedure) were higher in the EA group (two women; 0.82%) in comparison to the MA group (four women; 0.36%). The difference of 0.46% was not statistically significant as calculated by a Fisher’s exact test (*p* = 0.646) and was confirmed by an odds ratio of 0.673 (CI: 0.134–4.036). Table 3 summarises the pregnancy outcomes.

In the MA cohort, four procedure-related miscarriages were identified. In three out of these four cases, amniocentesis became complicated by an intrauterine death a few days after the procedure. The fourth one became complicated because of chorioamnionitis with premature labour as a result. Two cases of procedure-related miscarriages were described in the EA cohort. Both times, a preterm prelabour rupture of the membranes was a likely cause. No procedure-related pregnancy loss was found in 713 (99.44%) and 240 (99.17%) cases. More information about the miscarriages is summarised in Table 4.

## 4. Discussion

This study discovered no significant difference in the procedure-related risk of miscarriage when EA was compared to MA. The difference between the two groups was only 0.46%, with an odds ratio of 0.673 (CI: 0.123–0.699), which is statistically not significant. Nevertheless, there are a few hypotheses that could explain the difference between the two groups.

First, both cohorts were compared at two different points in time. This might have been a disadvantage for the EA group because spontaneous miscarriages at 14 gestational weeks were included in procedure-related losses. On the contrary, in the MA group, they were not taken into account, as the procedure had not yet been carried out. Second, because of the retrospective design of this study, it was not possible to create two similar groups on all accounts. The baseline characteristics provided in Table 2 indicate that the two groups were similar in terms of mean maternal age, which studies have shown to be an important predictor of pregnancy losses [1,5]. However, a significant difference was seen in the indication for undergoing amniocentesis and genetic test results. There were noticeably more chromosomal abnormalities found in the EA cohort, as well as more ultrasound abnormalities, positive NIPT results and abnormal first-trimester screenings. All of these factors bear an increased risk of miscarriage of their own.

The results of this study differ from those of the Canadian Early and Mid-trimester Amniocentesis Trial (CEMAT), an RCT from 1998 conducted on 4374 women, which is considered the gold standard. A post-procedural spontaneous loss rate of 2.6% was observed in the EA cohort and 0.8% in the MA cohort [8]. However, the CEMAT study found an increased risk of fetal loss in a very early amniocentesis group, which was between the 11^+0^ and 12^+6^ gestational weeks. The study recommended that the advantages of amniocentesis sampling between 13 and 15 gestational weeks remain to be assessed.

Because of the results of the CEMAT study, it was decided to exclude from this study amniocenteses performed between 11 and 13 gestational weeks. However, 26 (0.67%) out of 3824 women in the study had the procedure between 12 and 13 weeks. One procedure-related miscarriage was detected. Unfortunately, it was impossible to comment on these results given the small sample size. Therefore, they were also excluded.

Since the daunting results of the CEMAT study were published, little research has been conducted on EA [1,2,5,6,7,8,9]. In this respect, the current study is innovative and encouraging. In addition, the strength of this study is that all procedures were performed by three operators having the same amount of experience, with each having done more than 100 amniocenteses. Studies have shown that the risk of miscarriage decreases with more extensive technical experience [1,3,4,5,6,10]. The study results are comparable to those found by Wilson et al. They also found a post-procedure-related miscarriage risk of 2.4% in the EA cohort and 3.3% in the MA cohort [14] and concluded that EA was a safe alternative to MA. However, they also stated that a bigger multicentric RCT was needed to verify these results.

The design of the current study is its biggest weakness. An RCT would have been a better study design for this trial, but it is ethically unacceptable to expose one group of pregnant women to a procedure with risk of miscarriage and not expose another group of pregnant women to the same risk. Also, the number of amniocenteses is declining because of the NIPT [10]. So, it would take a lot of time to recruit enough women. The study design also did not use a ‘healthy’ control group because most women who need amniocentesis have an indication to do so (e.g., an ultrasound abnormality, positive serum screening or family history of genetic disorders). Unfortunately, it is this group that inherently has a higher risk of spontaneous miscarriage. This would lead to an overestimation of the procedure-related risk of miscarriage, meaning the numbers found in this study are not a reflection of an average population.

The retrospective study design also did not take other confounders into account. We know that there are variables other than the timing of the procedure that contribute to the overall miscarriage rate: maternal factors (such as age, smoking, diabetes, hypertension, high body mass index and autoimmune diseases) and placental or uterine factors (including fibromas, oligohydramnios, multiple pregnancies, vaginal infection or blood loss) [5,15,16]. These confounders make it difficult to know whether the miscarriage was induced by the procedure itself or had a natural cause. These confounders remain to be assessed. The specific procedural variables can also be described in a prospective study design, since these could be correlated with more fetal losses and adverse pregnancy outcome. For example, the position of the uterus, presence of uterine fibroid, subjective assessment of amniotic fluid volume, number of needle insertions, difficulty of the procedure, transbladder or transplacental technique and the occurrence of membrane tenting. Literature showed that the latter was associated with early amniocentesis [5,15,16].

There is also a discrepancy between the definitions of a procedure-related miscarriage. Some studies used a cut-off of 14 days, whereas others counted until 28 gestational weeks. It is likely that more pregnancy losses can be found when follow-up is increased. As the literature indicates that most miscarriages occur within 4 weeks of the procedure, this time period has been chosen as the cut-off in this study [2].

Regarding the use of microarray as the method of choice to analyse the amniocentesis samples, the eight Centers for Medical Genetics in Belgium agreed on national consensus guidelines on the use of genomic array in prenatal diagnosis in 2013 [17]. These guidelines regard the practical recommendation on the organization of pre- and post-counselling and how to interpret and report prenatal array results. They are regularly evaluated and adapted by a national committee, and offer a nation-wide homogenous policy concerning prenatal diagnosis. This represents a unique approach worldwide, and the decision to implement genomic array in prenatal diagnosis in Belgium reflects the potential of this technique to fulfil the longstanding need for a diagnostic test with a higher resolution and thus, yield than conventional karyotyping. Furthermore, it enables direct DNA extraction, and thus makes the need for time-consuming and expensive cell culture redundant.

## 5. Conclusions

The findings of this multicentre, retrospective cohort study indicate that amniocentesis performed early, at 14^1–6^ weeks, could be an alternative to the mid-trimester procedure done at 15^1–6^–17^1–6^ weeks. It might be considered a reliable alternative that shortens the time period between screening and diagnostic tests for aneuploidy, thus reducing parents’ psychological distress in this uncertain period. However further investigation with a larger sample size is necessary, preferentially in a multicentre prospective study design.

## Figures and Tables

**Table 1:** Gestational age (weeks + days).

	MA (*n *= 809) (%)	EA (*n* = 298) (%)
14 + 0		32 (10.74)
14 + 1		25 (8.39)
14 + 2		34 (11.40)
14 + 3		35 (11.74)
14 + 4		53 (17.79)
14 + 5		60 (20.13)
14 + 6		59 (19.80)
15^0^–15^6^	490 (60.57)	
16^0^–16^6^	228 (28.18)	
17^0^–17^6^	91 (11.25)	
Mean gestational age	15 + 6	14 + 4

EA, early amniocentesis; MA, mid-trimester amniocentesis.

**Table 2:** Patient characteristics.

		MA (%)	EA (%)
Genetic results	Normal	720 (89.00)	238 (79.87)
	Trisomy 21	46 (5.69)	38 (12.75)
	Trisomy 13	1 (0.14)	1 (0.34)
	Trisomy 18	5 (0.62)	2 (0.67)
	Others	37 (4.57)	19 (6.36)
Indication of amniocentesis	Ultrasound abnormality	134 (16.56)	58 (19.46)
	Positive serum screening	247 (30.53)	67 (22.48)
	Abnormal first-trimester screening	48 (5.93)	23 (7.72)
	Genetic disorder in family history	36 (4.45)	13 (4.36)
	Genetic disorder in previous pregnancies	52 (6.43)	16 (5.37)
	Maternal age	212 (26.21)	76 (25.50)
	Personal request	40 (4.94)	7 (2.35)
	Abnormal NIPT	34 (4.20)	34 (11.41)
	Inconclusive NIPT	5 (0.62)	2 (0.67)
	Abnormal CVS	1 (0.14)	2 (0.67)
Age (years)	<35	414 (51.17)	151 (50.67)
	≥35	395 (48.83)	147 (49.33)

EA, early amniocentesis; MA, mid-trimester amniocentesis; NIPT, non-invasive prenatal test; CVS, chorionic villus sampling.

**Table 3:** Primary outcomes.

		MA (%)	EA (%)
Procedure-related risk of miscarriage (<4 weeks)	Yes	4 (0.36)	2 (0.82)
	No	713 (99.44)	240 (99.17)
Non-procedure-related risk of outcome	Live births	705 (98.33)	239 (98.76)
	Miscarriage >4 weeks after the procedure	8 (0.11)	1 (0.41)
Total		717	242
Therapeutic abortions		92	56

EA, early amniocentesis; MA, mid-trimester amniocentesis.

**Table 4:** Procedure-related outcomes.

	Gestational Age	Indication AC	Result AC	Subjective Procedure Information	Miscarriage
MA1	17w1d	Ultrasound abnormality	Normal	Easy Procedure	MUI not further specified
MA2	15w2d	Abnormal first trimester screening	Normal	Not described	MUI not further specified
MA3	15w5d	Ultrasound abnormality	Normal	Uncomplicated procedure	MUI not further specified
MA4	15w0d	Abnormal First trimester screening	Normal	Easy procedure	Chorioamnionitis with premature labor
EA1	14w3d	Maternal Age	Normal	Not described	PPROM—Anhydramnion
EA2	14w1d	Maternal Age	Normal	Not described	Vaginal blood loss—PPROM

EA, early amniocentesis; MA, mid-trimester amniocentesis; NIPT, non-invasive prenatal test; AC, amniocentesis; MUI, mors in utero; PPROM, premature prelabour rupture of membranes.

## Data Availability

The data presented in this study are not publicly available due to privacy issues.
